# The Regulating Effect of CII-3 and Its Active Components from *Periplaneta americana* on M1/M2 Macrophage Polarization

**DOI:** 10.3390/molecules27144416

**Published:** 2022-07-10

**Authors:** Jinglei Xu, Yihao Che, Xinyue Liu, Chaohe Liu, Di Meng, Xiuqin Pang, Miao He, Guangming Liu, Chenggui Zhang, Dasong Yang, Huai Xiao

**Affiliations:** 1 Yunnan Provincial Key Laboratory of Entomological Biopharmaceutical R&D, Dali University, Dali 671000, China; xjl15093105827@163.com (J.X.); cheyihao1995@163.com (Y.C.); liuxyue1@163.com (X.L.); lch412728@163.com (C.L.); m18768891906@163.com (D.M.); p18787230348@163.com (X.P.); mio_h@163.com (M.H.); lgm13330555378@126.com (G.L.); chenggui_zcg@163.com (C.Z.); 2 CAS Key Laboratory of Tropical Marine Bio-Resources and Ecology, South China Sea Institute of Oceanology, Chinese Academy of Sciences, Guangzhou 510301, China; 3 University of Chinese Academy of Sciences, Beijing 101408, China; 4 School of Pharmacy, Shanxi Medical University, Taiyuan 030001, China

**Keywords:** *Periplaneta americana*, chemical components, pericanaside (**5**), M2 macrophages

## Abstract

CII-3 is the effective part of *Periplaneta americana* for application in oncotherapy. This study investigated its main chemical components for macrophage polarization regulation activity. Compounds were separated and purified, and their structures were elucidated based on NMR and HR-ESI-MS analyses. After inducing the M1 and M2 phenotype macrophages, CII-3 and testing components were added and co-incubated to evaluate their effects on the relevant markers of macrophages. Then, gradient concentrations of CII-3 and active monomers were further investigated for their effects on M2 macrophages. The effects were detected by RT-PCR, ELISA, flow cytometry, and immunofluorescence. Twelve compounds were identified from CII-3. CII-3 and pericanaside (**5**) had no obvious effect on M1 macrophages, while they significantly reduced the expression levels of M2 macrophage markers. Specifically, they significantly reduced the levels of TGF-β and IL-10 and the mRNA expression levels of ARG-1 and CD206 in the M2 phenotypes of RAW264.7 and Ana-1 macrophages. The conditioned medium of CII-3 and pericanaside (**5**) could inhibit the migration capacity of CT26.WT tumor cells. Macrophage M1/M2 polarization is a dynamic equilibrium, and the M2 phenotype, which can promote the growth of tumor cells, is relatively highly expressed in the tumor microenvironment. CII-3 and pericanaside could significantly reduce the phenotype of M2-type macrophages, indicating that the anti-tumor activity of CII-3 could be related to the inhibitory effect on M2 polarization, and pericanaside was one of the active components.

## 1. Introduction

Cancer is a significant disease affecting human health. Around 19.3 million tumor patients were predicted in 2020, and 10 million tumor-related cases of death were reported [1]. According to estimates, this considerable cancer burden will continually increase, and there may be 22 million newly diagnosed cancer patients and 13 million associated cases of death by the year 2030 [1,2].

An immune response is a natural defense mechanism within the body against tumors, and an imbalance in the immune effects is the key reason for tumor occurrence and progression. Macrophages are innate immune cells that have high plasticity and can perform different functions based on stimuli from the adjacent environment [3,4]. Macrophages are functionally categorized into the M1 and M2 phenotypes [5]. In the tumor microenvironment (TME), tumor-derived cytokines stimulate the macrophages into the M2 polarized phenotype, which can stimulate the migration and invasion of cancer cells by secreting various cytokines and chemokines [6]. Therefore, the inhibition of M2 polarization can effectively prevent tumor proliferation or metastasis.

Traditional Chinese medicine (TCM) has been found to be applied in clinical treatment for a long time [7]. TCM has general systematic and multi-target synergistic effects in the comprehensive treatment of malignant tumors, including immunomodulatory effects [8].

*Periplaneta americana* has been recorded in many authoritative TCM documents, such as Shennong’s Classic of Materia Medica [9]. With modern drug research, a series of Chinese patent medicines, such as “Kangfuxin Solution”, “Ganlong Capsule”, and “Xinmailong Injection”, were developed for use in the clinical setting [10]. The extract of *P. americana* demonstrated anti-tumor, immunity-enhancing, antibacterial, anti-inflammatory, analgesic, and tissue repair effects [11,12,13]. Modern pharmacological research showed that CII-3 was the active anti-tumor extract from *P. americana,* which could hinder tumor growth, enhance the immune capacity, and improve the survival status of experimental animals in several mouse tumor models [14]. In terms of immunomodulating activity, CII-3 could enhance the immune organ index and increase the number of peripheral blood immune cells in tumor-bearing mice, regulate the body’s immunological function, and act as an anti-tumor agent [15,16], and it could be a powerful candidate for treating tumors [17]. Further research showed that CII-3 could adjust the ratio of M1 and M2 in tumor-associated macrophages (TAMs), reducing M2 macrophages in vivo [18,19]. However, the compositions of CII-3, especially its active substances, remain unclear.

The current study illustrates the main chemical components of CII-3, and the immunomodulatory activities of these components on M1/M2 polarization. The M2 macrophage model was established by IL-4 inducing RAW264.7 or Ana-1 cells. Potential active samples were chosen through preliminary screening to identify those that could inhibit the mRNA expression of M2 markers ARG-1 and CD206 in IL-4-induced RAW264.7 cells by RT-PCR. The selected samples were further validated by RT-PCR, flow cytometry, immunofluorescence, and ELISA techniques. Finally, a transwell assay was used to detect the effects of active samples on the migration ability of tumor cells, namely, CT26 cells.

## 2. Results

### 2.1. Main Chemical Components of CII-3

**Figure 1:**
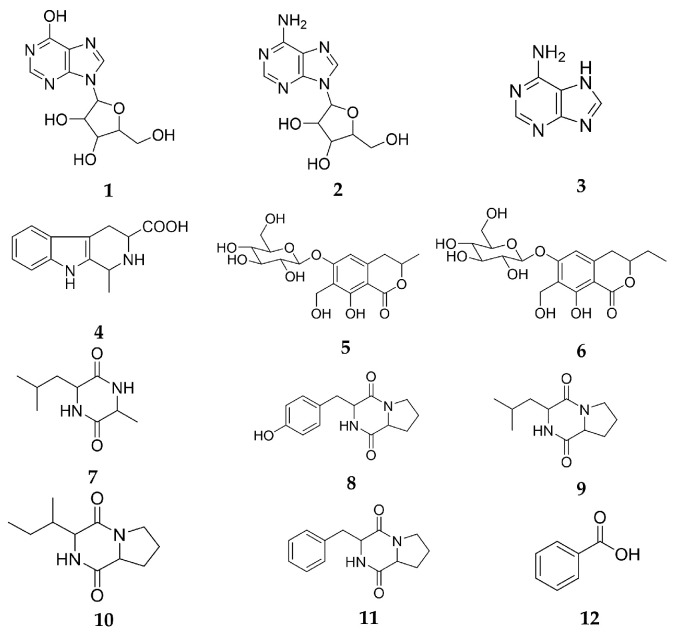
Structures of compounds 1–12.

Twelve compounds were obtained with the following structures: inosine (**1**) [20], adenosine (**2**) [21], adenine (**3**) [22], (-)-(1S, 3S)-1-methyl-1,2,3,4-tetrahydro-β-carboline-3-carboxylic acid (**4**) [23], pericanaside (**5**) [24], periplanoside B (**6**) [24], cyclo-(Leu-Ala) (**7**) [25], cyclo-(Pro-Tyr) (**8**) [26], cyclo-(Pro-Leu) (**9**) [27], cyclo-(Pro-Ile) (**10**) [23], cyclo-(Phe-Pro) (**11**) [23], and benzoic acid (**12**) [28]. They belonged to nucleosides, coumarin glycosides, cyclic peptides, and so on (Figure 1).

### 2.2. Primary Activity Screening of CII-3 and 12 Compounds on M1/M2 Macrophage Polarization

According to the MTT assay, CII-3 and its 12 compounds did not exert cytotoxicity below 150 µg/mL (CII-3)/30 µg/mL (compounds 1–12) on RAW 264.7 cells, and compounds 1–11 promoted the proliferation of these cells. In subsequent experiments, compounds 1–12 with a concentration of 30 μg/mL were selected for preliminary screening.

**Figure 2:**
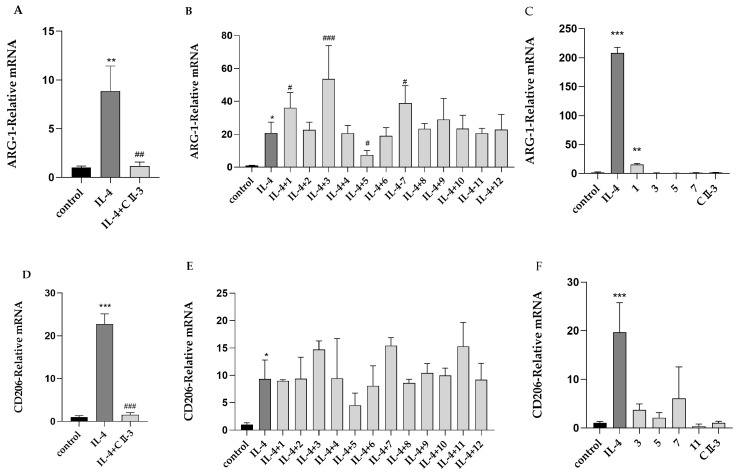
Expression effects of CII-3 and 12 compounds on ARG-1 and CD206 in IL-4-induced RAW 264.7 cells. ARG-1 expression affected by CII-3 (**A**), 12 compounds (**B**), and CII-3 and compounds (**1,3,5,7**) without IL4 pretreatment (**C**). CD206 expression effected by CII-3 (**D**), 12 compounds (**E**), and CII-3 and compounds (**3**,**5**,**7**,**11**) without IL-4 (**F**). * *p* < 0.05, ** *p* < 0.01, and *** *p* < 0.001 vs. the control group; ^#^ *p* < 0.05, ^##^ *p* < 0.01, and ^###^ *p* < 0.001 vs. the IL-4 group.

**Figure 3:**
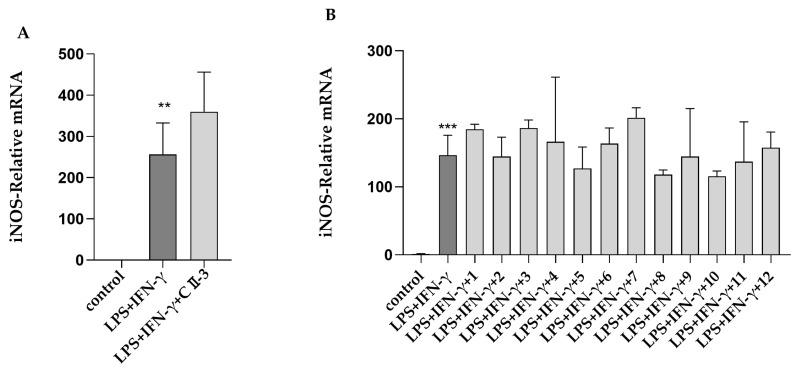
Expression effects of CII-3 and 12 compounds on iNOS in LPS and IFN-γ-induced RAW264.7 cells. iNOS expression affected by CII-3 (**A**) and 12 compounds (**B**). ** *p* < 0.01 and *** *p* < 0.001 vs. the control group.

The mRNA expression of the M2 markers CD206 and ARG-1 and M1 marker iNOS in the RAW 264.7 cells was assessed by RT-PCR. IL-4 dramatically enhanced the mRNA expression of the M2 markers CD206 and ARG-1 in RAW 264.7 cells relative to the uninduced group. The co-incubation of CII-3 with IL-4 decreased the mRNA expression of ARG-1 and CD206 (Figure 2A,D). In the M1 polarization model, the iNOS mRNA level significantly increased within the RAW 264.7 cells when LPS and IFN-γ were used for induction. CII-3 had no remarkable influence on the M1 phenotype macrophages (Figure 3A). The results supported the claim that CII-3 had an inhibitory effect on the polarization of M2 macrophages, and it did not damage the M1 phenotype macrophages [18].

The 12 compounds isolated from CII-3 were evaluated for their effects on M1/M2 macrophage polarization (Figure 2B,E). Compound **5** significantly inhibited ARG-1 mRNA expression (Figure 2B) and showed a decreasing tendency for the mRNA expression of CD206 (Figure 2E). Paradoxically, compounds **1**, **3**, and **7** could increase ARG-1 mRNA expression, and **3**, **7**, and **11** displayed rising trends for the mRNA expression of CD206. Thus, the direct influence of CII-3 and compounds **1**, **3**, **5**, **7**, and **11** on ARG-1 and CD206 mRNA expression in RAW 264.7 cells was tested again without IL-4 pretreatment (Figure 2C,F). The results showed that, with the exception of compound **1**, which could increase ARG-1 mRNA expression, other compounds (**3**,**5**,**7**,**11**) had no significant effect on the expression of either ARG-1 or CD206 mRNA (Figure 2C,F); in other words, compound 1 possibly had a promotional effect for M2 macrophage polarization, while compounds 3, 7, and 11 may have exerted a synergistic effect with IL-4 on RAW 264.7 cells. CII-3 and compound 5 had no effect on normal cells but could inhibit the IL-4-induced M2 polarization of RAW 264.7 cells. In the M1 polarization model, the 12 compounds had no significant effect on the mRNA expression of iNOS (Figure 3B).

### 2.3. The Influence of CII-3 and Pericanaside (5) on M2 Markers ARG-1 and CD206

Firstly, pericanaside (**5**) was not cytotoxic towards RAW 264.7 and Ana-1 cells at a concentration of 50 µg/mL, according to the MTT assay. Next, the effect of the three different concentrations of CII-3 and pericanaside (**5**) on the expression of various M2 markers was assessed in depth.

**Figure 4:**
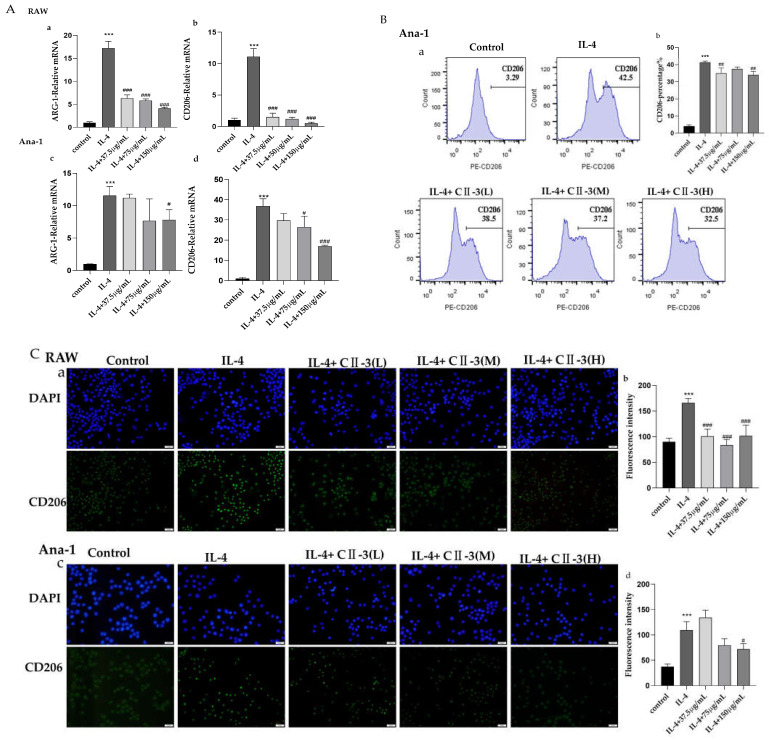
Impact of CII-3 on the expression of ARG-1 and CD206 in IL-4-induced RAW 264.7 and Ana-1 cells. (**A**): RT-PCR result of ARG-1 (**a**,**c**) and CD206 (**b**,**d**) in RAW 264.7 and Ana-1 cells; (**B**): Flow cytometry result of CD206 expression in Ana-1 cells (**a**,**b**); (**C**): Immunofluorescence result of CD206 expression in RAW 264.7 (**a**,**b**) and Ana-1 (**c**,**d**) (scale bar = 20 µm). *** *p* < 0.001 vs. the control group; ^#^ *p* < 0.05, ^##^ *p* < 0.01, and ^###^ *p* < 0.001 vs. the IL-4 group.

**Figure 5:**
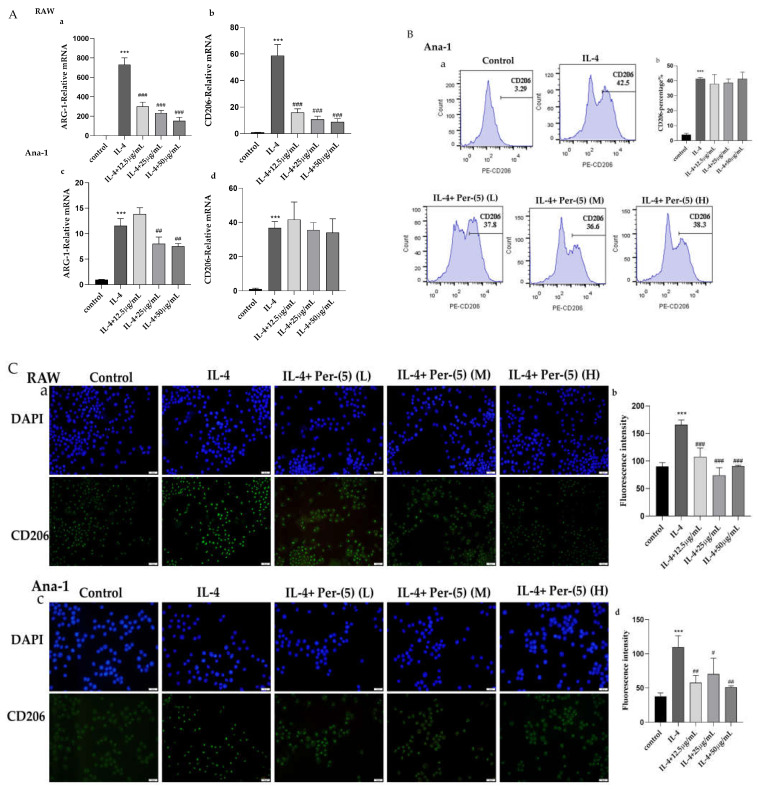
Impact of pericanaside (**5**) on the expression of ARG-1 and CD206 in IL-4-induced RAW 264.7 and Ana-1 cells. (**A**): RT-PCR result of ARG-1 (**a**,**c**) and CD206 (**b,d**) in RAW 264.7 and Ana-1 cells; (**B**): Flow cytometry result of CD206 expression in Ana-1 cells (**a**,**b**), (**C**): Immunofluorescence result of CD206 expression in RAW 264.7 (**a**,**b**) and Ana-1 (**c**,**d**) (scale bar = 20 µm). *** *p* < 0.001 vs. the control group; ^#^ *p* < 0.05, ^##^ *p* < 0.01, and ^###^ *p* < 0.001 vs. the IL-4 group.

RT-PCR was used to detect the effects of CII-3 and pericanaside (**5**) on the mRNA expression of the M2 markers ARG-1 and CD206. As shown in Figure 4A and Figure 5A, both in RAW 264.7 and Ana-1 cells, IL-4 treatment dramatically enhanced the mRNA expression of the M2 markers ARG-1 and CD206. CII-3 could remarkably downregulate the ARG-1 and CD206 expression in RAW 264.7 cells and in a dose-dependent manner (Figure 4Aa,b), while in Ana-1 cells, the effects were relatively weaker, and only the high dose (150 μg/mL) had downregulating effects on ARG-1, while the medium and high doses (75 μg/mL, 150 μg/mL) had downregulating effects on CD206 (Figure 4Ac,d). A similar expression pattern of ARG-1 and CD206 levels was found in RAW 264.7 cells induced by IL-4 in the groups treated with pericanaside (**5**) (Figure 5Aa,b). In Ana-1 cells, the medium and high doses (25 μg/mL, 50 μg/mL) of pericanaside (**5**) could inhibit the mRNA expression of ARG-1 but had no significant effect on the mRNA expression of CD206 (Figure 5Ac,d).

The impact of CII-3 and pericanaside (**5**) on the IL-4-induced M2 polarization of macrophages was further analyzed by flow cytometry (Figure 4B and Figure 5B). IL-4 enhanced the CD206 expression in Ana-1 cells, while in RAW 264.7 cells, all the IL-4 and the sample groups showed negative results. The flow cytometric analysis results revealed lower CD206 expression under high and low doses of CII-3 after co-incubation with IL-4, compared to the expression in IL-4-induced Ana-1 cells (Figure 4B). In comparison, the medium dose of CII-3 exerted a weak impact on CD206 expression (Figure 4B). However, pericanaside (**5**) had no obvious effect on the expression of CD206 (Figure 5B).

Immunofluorescence was used to further understand the expression and cellular localization of CD206 in the presence of CII-3 and pericanaside (**5**) (Figure 4C and Figure 5C). IL-4 substantially enhanced CD206 expression in RAW 264.7 and Ana-1 cells. CD206 was primarily localized in the cytosol. After the addition of CII-3 and pericanaside (**5**) to co-incubate with IL-4, the CD206 expression in RAW 264.7 was decreased (Figure 4Ca,b and Figure 5Ca,b). In Ana-1 cells, pericanaside (**5**) reduced the expression of CD206, while the effect of CII-3 on CD206 was relatively weak, and only the high dose had an inhibitory effect (Figure 4Cc,d and Figure 5Cc,d).

### 2.4. The Influence of CII-3 and Pericanaside (5) on TGF-β and IL-10

**Figure 6:**
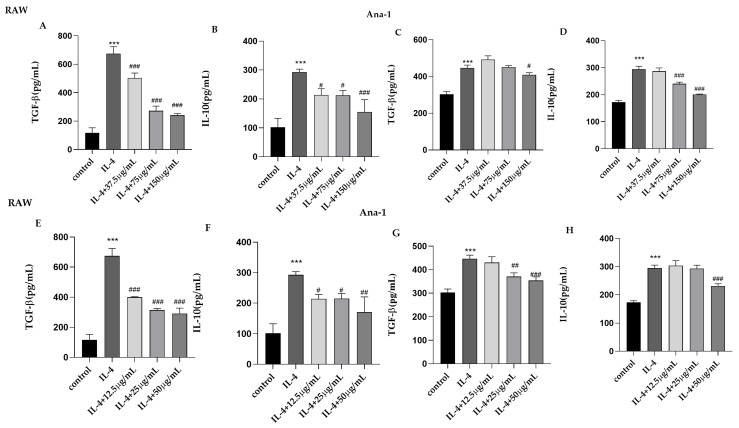
Effects of CII-3 and pericanaside (**5**) on the expression of TGF-β and IL-10 in IL-4-induced RAW 264.7 and Ana-1 cells. TGF-β (**A**) and IL-10 (**B**) expression in RAW 264.7 cells treated with CII-3; TGF-β (**C**) and IL-10 (**D**) expression in Ana-1 cells treated with CII-3. TGF-β (**E**) and IL-10 (**F)** expression in RAW 264.7 cells treated with pericanaside (**5**); TGF-β (**G**) and IL-10 (**H**) expression in Ana-1 cells treated with pericanaside (**5**). *** *p* < 0.001 vs. the control group; ^#^ *p* < 0.05, ^##^ *p* < 0.01, and ^###^ *p* < 0.001 vs. the IL-4 group.

In general, the levels of cytokines TGF-β and IL-10 were low in macrophages. After IL-4 treatment of RAW 264.7 and Ana-1 cells, the expression levels of TGF-β and IL-10 were significantly increased. After the addition of CII-3 to co-incubate with IL-4, the expression levels of TGF-β and IL-10 were significantly decreased in RAW 264.7 cells in a dose-dependent manner. In Ana-1 cells, the high dose of CII-3 (150 μg/mL) had a downregulating effect on the expression of TGF-β, and the medium and high doses of CII-3 (75 μg/mL, 150 μg/mL) had a downregulating effect on the expression of IL-10 (Figure 6). Additionally, a similar expression pattern of TGF-β and IL-10 levels was found in RAW 264.7 cells induced by IL-4 in the groups treated with pericanaside (**5**). Moreover, in Ana-1 cells, the medium and high doses of pericanaside (**5**) (25 μg/mL, 50 μg/mL) inhibited the expression of TGF-β, and the high dose of pericanaside (**5**) (50 μg/mL) reduced the expression of IL-10 (Figure 6).

### 2.5. The Influence of Conditioned Medium on CT26.WT Cell Migration by Transwell Assays

**Figure 7:**
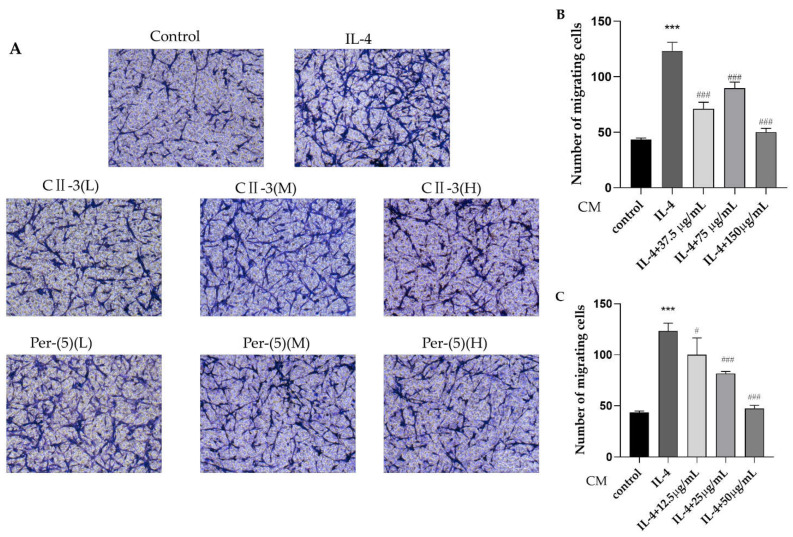
Impacts of CM on migration of CT26.WT cells. Transwell assays of CT26.WT cells following treatment with CM (**A**); number of migrating cells of CT26.WT treated with CM of CII-3 (**B**) and pericanaside (**5**) (**C**). *** *p* < 0.001 vs. the control group; ^#^ *p* < 0.05, and ^###^ *p* < 0.001 vs. the IL-4 group.

Transwell assays were adopted to evaluate in vitro cell migration. In line with the previous research of our research group, colon cancer CT26.WT was selected for the transwell assay, and the transwell assay results showed that treatment with IL-4-conditioned medium (CM) obviously enhanced the migratory ability of the cells when compared with the control group. Meanwhile, the CM of CII-3 could significantly reduce the number of migrating CT26.WT cells, and the CM of pericanaside (**5**) decreased the number of migrating CT26.WT cells in a dose-dependent manner (Figure 7).

## 3. Discussion

Cancer is an illness that poses a severe challenge to societies worldwide. Currently, no treatment method can completely inhibit tumor recurrence and metastasis [8].

Macrophages are essential innate immunity effector cells and significant components of the TME [29]. According to the polarization form, they adopt different phenotypes in response to microenvironmental changes and are categorized as M1/M2 macrophages [30]. The former type possesses pro-inflammatory and anti-tumor qualities, whereas the latter type is chiefly related to tissue growth and repair, with anti-inflammatory and tumor-promoting effects [31]. The TME contains massive amounts of TAMs, these mainly belong to the M2 phenotype and possess tumor-promoting effects. Therefore, suppressing the M2 phenotype could be an efficient cancer treatment strategy [32].

M2 macrophages express numerous anti-inflammatory molecules, including ARG-1, TGF-β, and IL-10, promoting tumor immunosuppression and progression [33]. ARG-1, the molecular marker of the M2 macrophages, is highly prevalent in cancer cells [34]. The level of ARG-1 is reduced in CD8^+^ T cells, thereby enhancing their anti-tumor activity. Therefore, M2 macrophages secrete ARG-1 to promote tumor growth and angiogenesis [35]. TGF-β aids in Treg cell differentiation to enhance cancer development, while IL-10 is an immunosuppressive factor, leading to the immune escape responses of tumor cells. CD206 is a particular M2 macrophage marker that significantly affects tumor cell growth and metastasis [36].

LPS and IFN-γ promote the release of inflammatory factors and, at the same time, initiate iNOS transcription, release NO, and trigger M1 macrophages’ polarization. RT-PCR was conducted with the purpose of detecting iNOS mRNA levels within the LPS and IFN-γ groups; the level was enhanced compared to the control group. CII-3 and pericanaside (**5**) had no significant effect on iNOS.

The impacts of CII-3 and pericanaside (**5**) on the M2 polarization of macrophages induced by IL-4 were analyzed. The M2 macrophage markers ARG-1, CD206, IL-10, and TGF-β induced by IL-4 were all reduced upon CII-3 and pericanaside (**5**) treatment. Moreover, the CM of CII-3 and pericanaside (**5**) inhibited the migration of colon cancer cells CT26.WT. These findings indicated that CII-3 and pericanaside (5) effectively suppressed M2 phenotypic differentiation in RAW264.7 and Ana-1 macrophages. The anti-tumor-migration effect of CII-3 and pericanaside (**5**) depends on the macrophage polarization. These data support the notion that CII-3 is a potent inhibitor of M2 activation, and pericanaside was found to be one of the active components. Additionally, compounds (**1**,**3**,**7**) combined with IL-4 had a synergistic effect and upregulated the expression of M2-type marker ARG-1 mRNA. This will be included in our next investigations.

Using RT-PCR, flow cytometry, and immunofluorescence technology, we detected M2 marker CD206 at the same time, but the results were different. RT-PCR is a sensitive method for mRNA detection, which detected macrophage M2 marker CD206 mRNA expression at the gene level, while flow cytometry detected the M2 marker CD206′s positive cell ratio at the protein level. The detection methods were different, and there may be small differences in the results.

Comparing the experimental results of flow cytometry and immunofluorescence, the degree of inhibition of CD206 expression by CII-3 and pericanaside (**5**) was different. Unfortunately, after our many experiments, the same treatment method was used in RAW264.7 cells as in Ana-1 cells; however, IL-4-induced RAW264.7 cells still could not be successfully stained with CD206 antibodies. There are also works similar to this study that failed to demonstrate successful staining [37]. There may be several reasons for this: firstly, RAW264.7 cells are very easily affected by the external environment and are activated to grow antennae, resulting in unsuccessful IL-4 induction. Secondly, an improper permeabilization time or choice of permeabilization reagent can result in the failure of CD206 antibody staining, demonstrated through comparison of the experimental results in Ana-1 cells. Flow cytometry detects the distribution of cell population characteristics and requires a large number of cells, while immunofluorescence detects the internal structure information of cells and requires a small number of cells. In comparison, the results of flow cytometry are more objective.

M2 macrophages can be obtained from IL-4-treated RAW264.7 and Ana-1 cells. After CII-3 and pericanaside (**5**) treatment, different methods were used to detect the expression of M2 macrophage markers. Although the general trend was inhibition, the degree of inhibition of macrophage polarization between the two cell lines was also different. This may be related to the source of the two mouse macrophages and the cell phenotype. Compared with RAW264.7 cells, Ana-1 cells are significantly less sensitive to stimulatory factors, and the specific mechanism needs to be further studied [38].

In this paper, the effects of the active extract CII-3 of *P. americana* and its active ingredient, pericanaside, on M2 macrophages were only investigated through in vitro cell models. However, the mechanism of inhibition of the polarization of M2 macrophages and the substances in the CM that exert anti-tumor cell migration are still unclear. It is necessary to continue to study the mechanism through in vitro models, and to further verify and explore it in vivo.

## 4. Materials and Methods

### 4.1. Materials

#### 4.1.1. Reagents

CII-3 was produced by Kunming SINOWAY Natural Pharmaceuticals Co., Ltd., Kunming, China, provided by Professor Liu Guang-ming of Dali University.

RAW 264.7 cells, DMEM, and RPMI 1640 were procured from Procell Life Science & Technology Co., Ltd. Wuhan, China. In addition, the Ana-1 cell line was obtained from Professor Yang Guo-ping of Dali University. The following reagents were utilized in the study: lipopolysaccharide (LPS) (Solarbio Science and Technology Co., Ltd., Beijing, China); Interferon-γ (IF-γ) (Peprotech Inc., East Windsor, NJ, USA); Interleukin-4 (IL-4) (Peprotech Inc., NJ, USA); MTT (Solarbio Science and Technology Co., Ltd.); RNeasy Isolation Reagent method, HiScript II qRT SuperMIX for qPCR (Vazyme Biotech Co., Ltd., Nanjing, China), ChamQ Universal SYBR qPCR Master Mix (Vazyme Biotech Co., Ltd., Nanjing, China); ELISA kits (Shanghai Enzyme Biotechnology Co., Ltd., Shanghai, China); PE anti-mouse CD206 antibody (BioLegend, San Diego, CA, USA) used for flow cytometry detection; CD206 antibody (Affinity Biosciences, Cincinnati, OH, USA) used for immunofluorescence; FITC-conjugated goat Anti-Rabbit IgG (H+L) (Affinity Biosciences, Cincinnati, OH, USA); DAPI (Solarbio Science and Technology Co., Ltd.); as well as PCR primers (Bioengineering (Shanghai) Co., Ltd., Shanghai, China)

#### 4.1.2. Device

The NMR spectra were recorded on a Bruker AV-400 spectrometer (Karlsruhe, Germany), with TMS being the internal reference for calibration. The detection of mass spectra was performed using the Agilent LC/MSD TOF (G3250AA) spectrometer. A cooling water circulation device (EYELA World, Tokyo, Japan), Sephadex LH-20 (GE Healthcare, Chicago, IL, USA), as well as a semi-preparatory HPLC system (Waters, P270 II, USA) were used in column chromatography. We carried out flow cytometry on a BD FACSC anto II (BD Biosciences, San Jose, CA, USA) flow cytometry system. The Multi-Mode microplate readers from Molecular Devices (SpectraMax M2, California, IL, USA) were utilized for MTT; the Bio-Rad CFX Connect Real-Time PCR System (Bio-Rad Laboratories, Hercules, CA, USA) was used for RT-PCR. In addition, immunofluorescence was performed on an Olympus microscope (BXS3, Tokyo, Japan).

### 4.2. Methods

#### 4.2.1. Isolation and Identification

CII-3 was prepared as in a patented method [39]; the crushed, air-dried *P. americana* powder was extracted with ethanol and concentrated, and it was then cooled and the upper oil removed; the lower solution was separated with macroporous resin column chromatography and eluted with alcohol/water to obtain the active part, CII-3.

In brief, 60 g CII-3 was purified by semi-preparation HPLC with an ODS column (CH_3_OH: H_2_O, 5–100%) to obtain ten fractions (Fr.1–Fr.10). Furthermore, the fractions were separated and purified by silica gel column chromatography, recrystallized, and subjected to ODS column chromatography, with an aqueous gradient of Sephadex LH-20, to provide compounds **1** (315.7 mg), **2** (20.5 mg), **3** (14.6 mg), **4** (38.5 mg), **5** (92.3 mg), **6** (10.9 mg), **7** (12.0 mg), **8** (18.5 mg), **9** (2.8 mg), **10** (20.7 mg), **11** (12.6 mg), and **12** (6.7 mg).

The structures of the compounds were identified by HR-ESI-MS, ^1^H NMR and ^13^C NMR.

#### 4.2.2. M1/M2 Polarization Models for RAW 264.7 and Ana-1 Cells

RAW 264.7 and Ana-1 cells in the logarithmic growth phase were treated with 1 μg/mL of LPS and 20 ng/mL of IFN-γ for 24 h to induce M1 phenotype macrophages, and they were treated with 20 ng/mL IL-4 for 48 h to generate M2 phenotype macrophages. After the M1 and M2 phenotype macrophages were induced, CII-3 and its 12 compounds were added to evaluate their effects. The primary M1 marker was iNOS, and the M2 markers were ARG-1, CD206, and TGF-β, as well as IL-10 [40,41].

#### 4.2.3. Cell Viability Assay

The MTT assay was conducted in line with a previous report to test the cytotoxic activity of CII-3 and its 12 compounds on RAW 264.7 cells [42]. After adjusting the cell concentration to 10^5^ cells/mL, 100 μL cells was inoculated in 96-well plates. After 24 h, 200 μL medium containing CII-3 and the 12 compounds was replaced and the mixture incubated for 24 h. Subsequently, 20 μL solution of 5 mg/mL of MTT was added to incubate for 4 h. With the MTT solution being discarded, 150 µL of DMSO was supplemented and the mixture shaken for 10 min. In addition, the absorbance (OD) was recorded at 490 nm, with cell viability being measured.

#### 4.2.4. RT-PCR

The RT-PCR method was employed with the purpose of detecting the mRNA expression of ARG-1, CD206, and iNOS by slightly modifying a previous literature report [43]. RT-RCR detected the expression of ARG-1, CD206, and iNOS in IL-4/LPS+IFN-γ-induced macrophages by CII-3 and the 12 compounds; the IL-4/LPS+IFN-γ group was used as a model control group. Next, the expression of ARG-1 and CD206 in macrophages without IL-4 induction by CII-3 and compounds **1**,**3**,**5**,**7**,**11** was examined. The IL-4 group served as a positive control. The PCR analysis was performed by adopting the Bio-Rad CFX Manager software. The data were analyzed by the method of 2 ^–ΔΔCt^. The PCR primer sequence was shown in Table A1. 

#### 4.2.5. Enzyme-Linked Immunosorbent Assay

To detect TGF-β and IL-10, ELISA was performed [42]. RAW 264.7 and Ana-1 cells (10^5^ cells/mL) were inoculated into 12-well plates. The culture supernatant of the cells was gathered and centrifuged to eliminate microscopic debris. TGF-β and IL-10 were analyzed using ELISA kits.

#### 4.2.6. Flow Cytometry

The treated RAW 264.7 and Ana-1 cells were centrifuged, rinsed with PBS, and subjected to centrifugation at 3000 rpm for 5 min. After adjusting the cell concentration to 10^6^ cells/mL, the cells were suspended in 500 μL of 4% paraformaldehyde (PFA) and fixed for 20 min. They were then rinsed with PBS and centrifuged at 3000 rpm for 5 min. Next, the cells were exposed to 0.1% of Triton X-100 suspension for 10 min, centrifuged at 3000 rpm for 5 min, and washed with PBS; cells were blocked with 200 μL of 3% BSA for 45 min. Next, the cells were nurtured with the PE anti-mouse CD206 antibody for half an hour. Finally, the cells underwent flow cytometry by adopting the BD FACSC anto II flow cytometry system, and the FlowJo software was used to analyze the results.

#### 4.2.7. Immunofluorescence

After placing the cell slides in 12-well plates, RAW 264.7 and Ana-1 cells (10^5^ cells/mL) were inoculated in 12-well plates. Later, cells growing onto the coverslips were fixed with 4% PFA and permeabilized for 15 min using 0.5% of Triton X-100 contained in PBS. Afterwards, the cells were blocked using 5% bovine serum albumin in PBS, stained with the primary antibody CD206, and later blotted with fluorescence-conjugated secondary antibodies. Subsequently, DAPI was adopted to mark the nuclei. The images were analyzed using the Olympus BXS3 microscope. Based on the image analysis program Image J, fluorescence intensity was explored in three random fields of view per section for quantitative analysis.

#### 4.2.8. Conditioned Medium

The RAW264.7 cells were incubated with IL-4 and CII-3/pericanaside (**5**) alone or together for 48 h; the supernatant was discarded and rinsed twice with PBS. Cells were cultivated with serum-free medium. After 24 h, the supernatants of the treated cells were gathered and centrifuged at 12,000 × *g* for 5 min, and the collected supernatants were the conditioned medium (CM).

#### 4.2.9. Transwell Migration Assay

In the migration assay, 10% FBS and the above-mentioned CM were added in the lower chamber, and CT26.WT cells (200 μL/well, 10^5^ cells/mL) in serum-free medium were supplemented in the upper chamber. After 24 h, the CM in the upper chamber was discarded, and 500 μL of 4% PFA was supplemented in the lower chamber and fixed for 15 min. Cells were washed in PBS. The unmigrated cells were carefully erased, and 500 μL of 0.1% crystal violet was supplemented in the lower chamber for staining. Finally, the migration of cells at the bottom of the chamber was observed under a microscope, and three fields were randomly selected to capture photographs. The Image J software was used for statistical analysis.

### 4.3. Statistical Analysis

The results are presented as the mean ± SD. Data were explored by adopting one-way ANOVA on GraphPad Prism 8.0, and *p* < 0.05 denoted statistical significance.

## 5. Conclusions

CII-3, the antineoplastic effective part of *P. americana*, could significantly reduce the markers of IL-4-induced M2 phenotype in RAW264.7 and Ana-1 macrophages, including the levels of TGF-β and IL-10, as well as ARG-1 and CD206. In CII-3, 12 compounds were identified. Among them, compound 5, pericanaside, could also downregulate the M2 phenotype markers. With the exception of flow cytometry, the test results of RT-PCR, ELISA, and immunofluorescence indicated that pericanaside significantly decreased the levels of TGF-β and IL-10, as well as ARG-1 and CD206. Both the conditioned media of CII-3 and pericanaside (**5**) could inhibit the migration capacity of CT26.WT tumor cells. In conclusion, CII-3 has a significant regulatory effect on tumor-promoting M2-type macrophages, and pericanaside was confirmed to be one of the active components.

## Data Availability

The data presented in this study are available on request from the first author.

## Sample Availability

Samples of the compounds are available from the authors.

## Appendix A

**Table A1:** Primer sequences used for RT-PCR.

Gene	Primer Sequences (5-3)	GeneBank ID
iNOS	Forward: TTTGCTTCCATGCTAATGCGAAAG Reverse: GCTCTGTTGAGGTCTAAAGGCTCCG	NM_001313922.1
CD206	Forward: CATGAGGCTTCTCTTGCTTCTG Reverse: TTGCCGTCTGAACTGAGATGG	XM_021173919.1
ARG-1	Forward: CTCCAAGCCAAAGTCCTTAGAG Reverse: AGGAGCTGTCATTAGGGACATC	NM_008625.2
GAPDH	Forward: ATACGGCTACAGCAACAGGG Reverse: GCCTCTCTTGCTCAGTGTCC	NM_001289726.1
